# Evolution of 3'UTR-associated RNAs

**DOI:** 10.1186/1471-2105-16-S8-A7

**Published:** 2015-04-30

**Authors:** Jan Engelhardt, Peter F Stadler

**Affiliations:** 1 Bioinformatics Group, Department of Computer Science, University of Leipzig, Leipzig, Germany

## Background

Despite their abundance, unspliced EST data has received little attention as a source of information on non-coding RNAs. Very little is known, therefore, about the genomic distribution of unspliced non-coding transcripts and their relationship with the much better studied regularly spliced products. In particular, their evolution has remained virtually unstudied. A subclass of the unspliced EST cluster consists of so called 3'UTR-derived RNAs (uaRNAs). They can have functions in cis and trans, independent from the harboring gene. uaRNAs can be detected by combining EST data with predicted transcription start sites.

## Results

We systematically study the evidence on unspliced transcripts available in EST annotation tracks for human and mouse, comprising 104,980 and 66,109 unspliced EST clusters, respectively. 15-20\% of the unspliced EST cluster are conserved between human and mouse. More than 7,000 human and 6,000 mouse unspliced EST cluster overlap the 3'UTR of a RefSeq gene or are located within 5kb downstream of the 3' end. Using TSS predicted by chromatin data we identify a total of 1,547 bona fide uaRNA candidates in human. Integrating only the public available CAGE data by the FANTOM5 consortium we predict a total of 1,891 uaRNA candidates in human and 2,477 candidates in mouse.

We also give a first glimpse on the sequence and structure conservation of these uaRNA candidates.

## Conclusions

Expressed sequence tag data combined with experimentally predicted promoter data, e.g. CAGE, is a powerful tool to identify candidate uaRNAs. This combination of data sets could also be applied to non-model organisms without a sequenced genome. uaRNAs are a quite new class of non-coding RNAs and have not been extensively analysed yet. We present a catalog of candidates which are excellent targets for experimental verification. Increasing evidence hints to additional regulatory functions of 3'UTRs independent from the processing of the corresponding gene. It is very likely that this mechanism is not only present in humans and mice but also other eukaryotes. Using public data is a great way to get a glimpse of the uaRNAome of the respective species.

